# Mechanisms of peripheral sensitization in endometriosis patients with peritoneal lesions and acyclical pain

**DOI:** 10.1007/s00404-023-07110-9

**Published:** 2023-07-05

**Authors:** Renata Voltolini Velho, Jalid Sehouli, Sylvia Mechsner

**Affiliations:** Department of Gynecology Charité with Center of Oncological Surgery, Endometriosis Research Center Charité, Campus Virchow-Klinikum, Berlin, Germany

**Keywords:** Endometriosis, Nociceptive markers, Nociceptive receptors, Hormonal treatment, Pelvic pain

## Abstract

**Purpose:**

Endometriosis (EM) is one of the most frequent differential diagnoses concerning chronic pelvic pain. Women under hormonal therapy (HT) often benefit from it but sometimes suffer a setback and develop acyclical pelvic pain. Due to the assumption that mechanisms of neurogenic inflammation are involved in the generation of chronic pelvic pain, we aimed to investigate the expression of sensory nerve markers in EM-associated nerve fibers of patients with/without HT.

**Methods:**

Laparoscopically excised peritoneal samples from 45 EM and 10 control women were immunohistochemically stained for: PGP9.5, Substance P (SP), NK1R, NGFp75, TRPV-1, and TrkA. Demographics and severity of pain were documented.

**Results:**

EM patients showed a higher nerve fiber density (PGP9.5 and SP) and increased expression of NGFp75, TRPV1, TrkA, and NK1R in blood vessels and immune cells compared with controls. Patients with HT have cycle-dependent pelvic pain but suffer from acyclical pelvic pain. Interestingly, reducing NK1R expression in blood vessels under HT was observed. A correlation between dyspareunia severity and nerve fibers density and between NGFRp75 expression in blood vessels and cycle-dependent pelvic pain severity was observed.

**Conclusion:**

Patients under HT have no ovulation and no (menstrual) bleeding, which correlate with inflammation and cyclical pain. However, acyclical pain seems to be due to peripheral sensitization once it is present under treatment. Neurotransmitters, like SP and their receptors, are involved in mechanisms of neurogenic inflammation, which are relevant for pain initiation. These findings indicate that in both groups (EM with/without HT), neurogenic inflammation is present and responsible for acyclical pain.

**Supplementary Information:**

The online version contains supplementary material available at 10.1007/s00404-023-07110-9.

## What does this study add to the clinical work

**Table:** 

Endometriosis (EM) is one of the most frequent differential diagnoses concerning chronic pelvic pain. Our findings indicate that in EM patients with and without hormonal therapy, neurogenic inflammation is present and responsible for acyclical pain.	

## Introduction

Characterized by the ectopic deposition and growth of endometrial-like tissues, endometriosis (EM) is an estrogen-dependent and inflammatory disorder [1]. EM lesions infiltrate adjacent organs (e.g., genitals, bladder, intestine, abdomen), resulting in inflammation, formation of scar tissue, and functional impairments of affected organs [1, 2]. The symptoms often affect patients’ psychological and social well-being and impose a substantial economic burden on society. For this reason, EM is considered a disabling condition that may significantly compromise social relationships, sexuality, and mental health [3–5]. Approximately 10% of women of reproductive ages are affected, i.e., 2 million women in Germany and 270 million worldwide, and 30–50% of them suffer from infertility [6]. Despite its negative impact on the quality of a patient’s life, many issues related to EM remain unclear.

Due to the duration of pain and dissemination of endometriotic lesions, the associated symptoms showed a wide variation including cyclic and acyclic lower abdominal pain, dysmenorrhoea, dyspareunia, dyschezia, dysuria and sub- or infertility. The pathogenesis of pain generation is very complex [1, 7, 8]. Recent evidence demonstrates that the peripheral nervous system plays an important role in the pathophysiology of this disease. Our group focus on understanding pain generation due to peritoneal lesions and already demonstrated wide changes in the innervation in the EM-affected peritoneum [9]. The higher density of sensory nerve fibers, the lower density of sympathetic nerve fibers, the release of proinflammatory neurotransmitters like SP and CGRP as well as periendometriotic inflammation suggested neurogenic inflammatory reaction in this tissue. Peripheral sensitization of EM-associated nerve fibers might be one key player in the modulation and severity of pain [10].

Because the pathogenesis of EM is still unresolved, no causal treatment options are available. The primary treatment goals are to relieve pain and eliminate fertility issues in women who wish to conceive [11]. Hormonal therapy is the first line of treatment for women with EM [12]. This treatment decreases the production of the estrogen-induced release of prostaglandins and consequently inflammation [13]. With continuous hormonal treatment, dysmenorrhea may be reduced compared to cyclic use, but the incidence of erratic bleeding may increase, and safety issues have not been fully studied [12]. Also, the development of acyclical pain under hormonal treatment is possible and described. We observed a high grade of inflammation especially in peritoneal lesions of symptomatic patients under hormonal treatment [7]. Taking together, we aimed to understand this pathway of pain generation in more detail and investigate the expression of sensory nerve markers in the periphery of peritoneal EM under the influence of hormonal therapy. The characteristics of tissue were also analyzed concerning the acyclic pelvic pain experience of EM patients.

## Methods

### Patients

This prospective study enrolled 55 women. Forty-five EM patients, who underwent laparoscopy due to symptomatic EM with excision of endometriotic lesions, were included. The diagnosed EM was staged according to the revised classification of the American Society of Reproductive Medicine (rASRM) as I: minimal, II: mild, III: moderate, and IV: severe. In the analysis, two stages had been considered: mild (rASRM I and II) and severe (rASRM III and IV). Ten control samples were collected from women without EM, who had undergone laparoscopy for benign gynaecological presentations such as nonendometriosis associated with ovarian cysts, uterine fibroids, hydrosalpinx, pelvic pain, peritonealized tissue or the unfulfilled wish to have children.

Patients were selected based on clinical intraoperative and subsequent histopathologic findings. All patients had been given a complete gynaecological examination. The severity of pain was documented using a standardized questionnaire with a visual analog scale (VAS). The pain intensity was determined with the help of a visual numerical analog scale (0 = no pain, 10 = strongest imaginable pain). The women were divided into two groups based on the pain scale: moderate pain (0–5 on the scale) and severe pain (6–10 on the scale).

The study was approved by the Institutional Review Board of the Charité University Medical Centre (Ethic vote EA4/036/12). All patients gave their consent.

### Sample collection and immunohistochemistry of peritoneal endometriotic lesions processing

All the surgically excised lesions (EM patients) and healthy peritoneum (control samples) were immediately fixed in buffered formalin 4% for 12 h and thereafter embedded in paraffin. Two μm thickness sections were immunohistochemically stained with antibodies (Supplemental Table I) against the nerve fibers markers: protein gene product 9.5 (PGP9.5), Substance P (SP); and nociceptive receptors: Neurokinin-1 Receptor (NK1R), Nerve Growth Factor Receptor p75 (NGFp75), Transient Receptor Potential Vanilloid 1 (TRPV-1), and Tropomyosin Receptor Kinase A (TrkA).

Negative control sections were processed by omitting the specific primary antibody. A skin incision and a tissue section of peritoneal EM with large nerve incisions were used as the positive control. Staining was detected using an axiophot (Carl Zeiss, Göttingen, Germany) microscope. Photomicrographs were taken at different magnifications (100 and 400) and were further processed using Adobe Photoshop (Adobe Systems, Unterschleissheim, Germany).

### Determination of nerve fiber density

The density of PGP9.5 and SP-positive nerve fibers was assessed by counting the number of immunostained nerves proximal to the endometriotic lesions (epithelial, stromal, and smooth muscle cells) and in the distal area at 1 mm^2^.

The “hotspot” method [14] was used to determine the nerve fiber density of the control tissue. The immunostained section was scanned at low magnification (10 ×), and the tissue area with the greatest number of nerves (“hotspot”) was selected. Five hotspots were evaluated and averaged for each control. The density was measured by the sequential assessment of two investigators. In cases of discrepant results, both observers repeated the analysis together and reached a consensus.

### Statistical analysis

Statistical analysis was performed using Graphpad Prism 9 and non-parametric (Mann–Whitney-*U*, Wilcoxon, Kruskal–Wallis or Spearman correlation test). *χ*^2^ and Fisher’s exact tests were used for the qualitative variable. Statistically significance was assumed for *p* < 0.05.

## Results

### Patients’ characteristics

Demographic and clinical variables for the 55 women recruited for this study are summarized in Table 1. Our case group comprised 45 EM patients, 20 (44.44%) presented minimal to mild EM (rASRM I and II) and 25 (55.56%) moderate to severe (rASRM III and IV). Twenty-three (23/45) of them were under hormonal therapy at the time of the surgery. Of these 23, 8 women received progestin-only therapy, 10 a combined progestin–estrogen, and in 5 EM patients, the preparation taken could no longer be determined. The mean age of the EM patients was 31.1 (19–53) years. EM patients who took hormones were on average younger (28.1) than women who did not take hormonal preparations (34.3; *p* = 0.0037).

**Table 1:** Characteristics of the study population

Group		Nr	Age	rASRM	Hormonal therapy	Pain characterization (pain intensity)						
						Pelvic pain	Cycle-dependent pelvic pain	Cycle-independent pelvic pain	Dysmenorrhea	Dyspareunia	Dyschezia	Dysuria
EM patients	H−	1	35	Severe	–	Yes	NA	NA	Yes (NA)	NA	NA	NA
		2	33	Mild	–	Yes	Yes (3)	No	Yes (3)	Yes (2)	No	No
		3	32	Severe	–	Yes	Yes (NA)	NA	Yes (3)	Yes (10)	No	No
		4	39	Mild	–	Yes	Yes (2)	NA	Yes (3)	Yes (NA)	Yes (NA)	NA
		5	43	Severe	–	Yes	NA	NA	Yes	NA	NA	NA
		6	25	Severe	–	Yes	Yes (8)	Yes (6)	Yes (7)	Yes (8)	No	No
		7	29	Severe	–	Yes	Yes (5)	No	Yes (7)	Yes (1)	No	No
		8	36	Severe	–	Yes	Yes (5,5)	Yes (7,5)	Yes (5,5)	Yes (4,5)	No	No
		9	53	Severe	–	Yes	NA	NA	Yes (NA)	NA	Yes (NA)	NA
		10	27	Severe	–	Yes	Yes (3)	No	Yes (3)	Yes (3)	Yes (3)	No
		11	32	Severe	–	Yes	Yes (5)	No	Yes (7)	Yes (2)	Yes (6)	No
		12	42	Severe	–	Yes	NA	NA	NA	No	Yes (NA)	No
		13	26	Severe	–	Yes	NA	NA	Yes (NA)	Yes (NA)	Yes (NA)	Yes (NA)
		14	30	Severe	–	NA	NA	NA	NA	NA	NA	NA
		15	38	Severe	–	Yes	Yes (5)	Yes (2)	Yes (4)	Yes (2)	Yes (4)	No
		16	29	Mild	–	Yes	NA	NA	Yes (NA)	Yes (NA)	No	Yes (NA)
		17	28	Mild	–	Yes	Yes (6)	Yes (8)	Yes (8)	Yes (3)	No	Yes (5)
		18	22	Severe	–	Yes	Yes (9)	Yes (8)	Yes (9)	Yes (9,5)	No	Yes (6)
		19	42	Severe	–	Yes	NA	NA	Yes (NA)	NA	NA	NA
		20	38	Mild	–	Yes	No	No	Yes (3)	Yes (2)	Yes (5)	No
		21	27	Mild	–	Yes	NA	NA	NA	NA	NA	NA
		22	49	Severe	–	NA	NA	NA	No	NA	NA	NA
	H +	23	22	Mild	E, POP	No	NA	NA	NA	NA	NA	NA
		24	26	Mild	COC	Yes	No	Yes (6)	Yes (0)	No	No	No
		25	19	Mild	COC	Yes	NA	NA	Yes (NA)	Yes (NA)	Yes (NA)	NA
		26	35	Mild	COC	Yes	Yes (7)	Yes (7)	Yes (8)	Yes (7)	Yes (5)	No
		27	28	Severe	NA	Yes	NA	NA	Yes (NA)	Yes (NA)	No	No
		28	35	Mild	POP	Yes	NA	NA	Yes (NA)	NA	NA	NA
		29	26	Mild	NA	Yes	NA	NA	Yes (NA)	No	NA	Yes (NA)
		30	23	Mild	POP	Yes	NA	NA	Yes (NA)	Yes (NA)	NA	NA
		31	30	Severe	POP	Yes	Yes (5)	No	Yes (7)	Yes (3)	Yes (7)	No
		32	22	Severe	POP	Yes	No	Yes (4)	Yes (6)	Yes (7)	Yes (8)	Yes (5)
		33	39	Mild	COC	Yes	NA	NA	Yes (NA)	Yes (NA)	No	NA
		34	32	Mild	POP	Yes	No	NA	Yes (6)	Yes (NA)	No	No
		35	36	Mild	COC	Yes	NA	NA	Yes (NA)	Yes (NA)	Yes (NA)	No
		36	27	Mild	NA	Yes	NA	NA	Yes (NA)	NA	NA	NA
		37	29	Severe	POP	Yes	No	NA	Yes (0)	Yes (5)	No	No
		38	23	Severe	COC	Yes	No	Yes (4)	Yes (10)	Yes (6)	Yes (8)	No
		39	25	Severe	COC	Yes	NA	NA	Yes (NA)	NA	No	No
		40	24	Severe	NA	Yes	Yes (NA)	Yes (5)	No	Yes (NA)	No	No
		41	27	Mild	NA	Yes	NA	NA	Yes (NA)	NA	Yes (NA)	NA
		42	30	Severe	COC	Yes	NA	NA	Yes (NA)	NA	NA	Yes (NA)
		43	24	Mild	POP	Yes	No	No	Yes (4)	No	Yes (3)	No
		44	35	Mild	POP	Yes	NA	NA	Yes (NA)	NA	NA	NA
		45	30	Severe	COC	Yes	Yes (8)	NA	Yes (8)	Yes (NA)	No	Yes (NA)
Control	H−	C1	26	–	–	Yes	NA	NA	No	NA	NA	NA
		C2	38	–	–	NA	NA	NA	NA	NA	NA	NA
		C3	45	–	–	NA	NA	NA	NA	NA	NA	NA
		C4	27	–	–	Yes	Yes (9)	No	Yes (3)	Yes (9)	No	No
		C5	28	–	–	No	NA	NA	Yes (NA)	Yes (NA)	NA	NA
		C6	27	–	–	Yes	NA	NA	NA	NA	NA	NA
	H +	C7	46	–	NA	No	NA	NA	No	NA	NA	NA
		C8	43	–	POP	Yes	NA	NA	NA	NA	NA	NA
		C9	20	–	COC	Yes	Yes (5)	Yes (2)	Yes (NA)	No	No	NA
		C10	21	–	COC	Yes	Yes (4)	Yes (3)	Yes (4)	Yes (4)	Yes (4)	Yes (2)

*H*+  hormonal therapy, *H*− no hormonal therapy, *mild* rASRM I and II, *severe* rASRM III and IV, *E* estrogen, *POP* progestogen, *COC* combined pills, *NA* no information

The control group was a compost of ten patients, four of them received hormonal therapy. In one case, it was a pure progestin therapy, in two others, a combined progestin–estrogen therapy and in one other case, the product ingested could no longer be determined. Women in the control group were on average 32.1 (20–46) years old. No significant difference in age between the non-EM patients who took hormones (32.5) and the ones who did not take (31.8) was observed.

### Pain characterization

Forty-two EM patients (95.5%) reported pelvic pain. One patient under hormonal treatment reported no pelvic pain. In two cases, no statement was made, these women were not taking any hormones. In the control group, six patients (54.5%, 3 under hormonal therapy) stated to suffer from pelvic pain. Three (27.3%) denied suffering from this pain (one under hormonal therapy), and in two cases (18.2%), no information was given. All pain and patient characterization are summarized in Table 1.

#### Cycle-dependent pelvic pain (CDPP)

Of the 42 EM patients reporting pelvic pain, 15 (33.3%) communicate suffering from CDPP. Four of them were on hormonal therapy and eleven were not. A statistical difference (*p* = 0.0096) could be seen in the hormonal therapy and this pain. No CDPP was reported in seven EM patients (one without hormonal therapy). For 23 patients with EM (13 positives for hormone therapy), this information was missing. In the control group, three (27.3%) women reported CDPP. Two (18.2%) of them were on hormonal therapy. In seven cases (70%), this statement was missing.

Regarding the strength of the CDPP, EM patients (data from 13 women—10 negatives for hormonal therapy) suffered on average from a pain level of 5.5 (2–9). No difference in the average pain severity was found between EM patients who were on hormonal treatment (6.7; 4–7) and those who were not (5.1; 2–8). In the control group (data from three patients), the severity of the CDPP was an average of 6 (4–9) and did not differ from the group of EM patients.

#### Cycle-independent abdominal pain (CIAP)

Only ten (22.2%) EM patients describe suffering from CIAP with an average severity of 5.7 (2–8). Five EM patients under hormonal therapy report on average a CIAP of 5.2 (4–7) and for the five patients without hormonal therapy, a pain average of 6.3 (2–8). Two (20%) women under hormonal therapy from the control group also describe feeling this pain in severity 2 and 3.

#### Dysmenorrhea

Dysmenorrhea or painful bleeding in cyclical modus of combined pills (withdrawal bleeding) was a symptom communicated from 39 (86.7%) EM patients, with 21 under hormonal therapy. Two patients (4.4%—1 receiving hormonal treatment), said do not suffer from this and the other four (8.9%) did not answer. In the control group, dysmenorrhea was expressed as a symptom for four women (40%), two of these being on hormonal therapy. Two patients (20%, 1 taking hormones) affirmed no painful bleeding in the cyclical modus of combined pills. This information was missing for four patients (40%) in the control group, two under the hormonal treatment.

The severity of the dysmenorrhea or painful bleeding was informed for 21 EM patients (46.7%), 9 of them were under hormonal therapy. No statistical difference was found in the severity of the dysmenorrhea between EM patients who were under hormonal therapy (5.4; 0–10) and those who were not (5.2; 3–9). In the control group, two (20%) women reported the severity of this symptom. One patient who was taking hormones reported a pain level of 4 while the other, who was not taking hormones, reported a pain level of 3.

#### Dyspareunia

Twenty-seven (60%, 13 on hormonal therapy) EM patients reported dyspareunia. Four women (8.9%, 3 under hormonal treatment) stated no pain during sexual intercourse. This information was not given for 14 EM patients (31.1%, 7 under hormonal treatment). No information was given for six women (60%, two received hormones, four did not) from the control group. Three patients (27.3%, one taking hormones) suffered from dyspareunia. Only one control patient (10%), who was under hormonal therapy, affirmed not to have this pain.

EM patients reported an average degree of dyspareunia of 4.7 (1–10). Five patients, who were under hormonal therapy, reported a mean pain score of 5.6 (3–7). The other 11, who were not taking hormones, reported an average pain of 4.3 (1–10). Two patients from the control group (one patient in hormonal therapy) reported a pain score of 4 and 9.

#### Dyschezia

Dyschezia was indicated as a symptom in 16 (35.5%) patients with EM, 8 of them on hormonal therapy. Another 16 women (35.5%) stated that they did not have dyschezia. Of these, eight women took hormones. Thirteen EM patients (28.9%) did not answer this question. In the control group, one woman who received hormones suffered from dyschezia severity 4. Two women, one of them taking hormones, denied suffering from this condition (20%). In seven cases (70%, two did take hormones), the corresponding information was lacking in the questionnaire.

A mean pain severity of 5.4 (3–8) concerning dyschezia was given in 16 EM patients (35.6%). Eight patients who were on hormone therapy estimated pain intensity to be a mean of 6.2 (3–8). However, the other eight women who did not receive hormones reported an average pain of 4.5 (3–6).

#### Dysuria

Eight (17.8%) EM patients suffered from dysuria, four of them were on hormone therapy. In this group (only three patients answered this question), the average severity of the dysuria was given as 5.3 (5–6). In 21 cases (46.7%, 11 on therapy), the dysuric pain was denied and in 16 cases (35.6%), the information was missing. One control patient affirmed suffering from dysuria with a severity of 2, another denied it. Both were under hormonal treatment. In eight cases (80%), this information was missing.

### Immunohistochemistry results

**Fig. 1:**
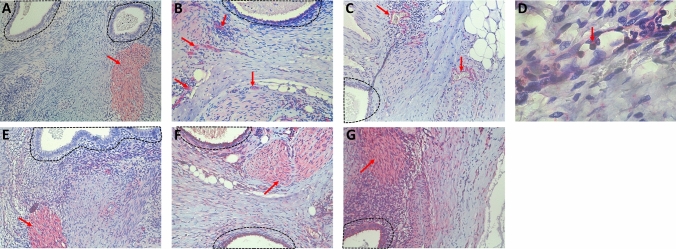
Expression of PGP9.5 (**A**), Substance P (**B**), NK1R-positive blood vessels (**C**), immune cells (**D**), NGFRp75 (**E**), TRPV1 (**F**), and TrkA (**G**) (red arrow) surrounding the endometriotic lesion (black marked) was counted. All pictures are in 400 × magnification, except **D**, which is in 1000 × magnification

All the results are presented as median, 25–75% percentile. Figure 1 shows the immunohistochemistry results as an example.

#### Nerve fibers density in endometriotic lesions

**Fig. 2:**
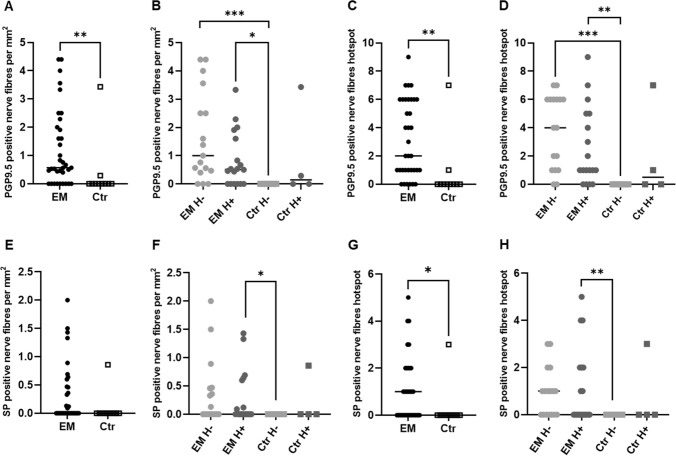
Nerve fibers density in endometriotic lesions and healthy peritoneum. PGP9.5-positive nerve fibers per mm^2^ (**A**–**B**) and hotspot (**C**–**D**). Substance P (SP)-positive nerve fibers per mm^2^ (**D**–**E**) and hotspot (**F**–**G**). *EM* endometriosis patients, *Crt* control, *H +*  under hormonal treatment, *H−* without hormonal treatment; all the results are presented as median, 25–75% percentile. Mann–Whitney test and Kruskal–Wallis with Dunn’s multiple comparison tests. *p < 0.05; **p < 0.01; ***p < 0.001

Using anti-PGP9.5 and anti-SP, nerve fibers were detected in peritoneal specimens from women with EM and healthy peritoneum from women without EM. PGP9.5 nerve fibers density was significantly increased in endometriotic lesions (0.57, 0.0–2.0) compared to healthy peritoneum (0.0, 0.0–0.072; *p* = 0.0079) both in the view of nerve fibers per mm^2^ and in the hotspot image (EM: 2.0, 1.0–6.0; Control: 0.0, 0.0–0.25; *p* = 0.0017) (Fig. 2A and C). EM patients showed significantly more SP-positive nerve fibers in the hotspot view (EM: 1.0, 0.0–2.0; control: 0.0, 0.0–2.0; *p* = 0.0393) but no difference in the density of SP-positive nerve fibers per mm^2^ (EM: 0.0, 0.0–0.47; control: 0.0, 0.0–0.86; *p* = 0.0874) (Fig. 2E and G).

When the hormonal therapy is taken into consideration, statistical significance is observed in the EM group with (EM H+) and without hormonal intake (EM H−) compared with the control without treatment (Ctr H−) for PGP9.5 both in the nerve fibers per mm^2^ (EM H + : 0.51, 0.0–1.7; *p* = 0.0123; EM H−: 1.0, 0.4–3.0; *p* = 0.0001; Ctr H−: 0.0, 0.0–0.0) and in the hotspot image view (EM H + : 1.0, 0.7–5.0; *p* = 0.0028; EM H−: 4.0, 1.0–6.0; *p* < 0.0001; Ctr H−: 0.0, 0.0–0.0) (Fig. 2B and D).

No correlation could be seen between the nerve density and the rASRM stages. The PGP9.5 (hotspot view, *r* = 0.728; *p* = 0.0029) and SP (hotspot and nerven/mm^2^, *r* = 0.5741; *p* = 0.0278 and *r* = 0.7118; *p* = 0.004, respectively) nerve fibers density correlated with dyspareunia pain levels (Table 2).

**Table 2:**
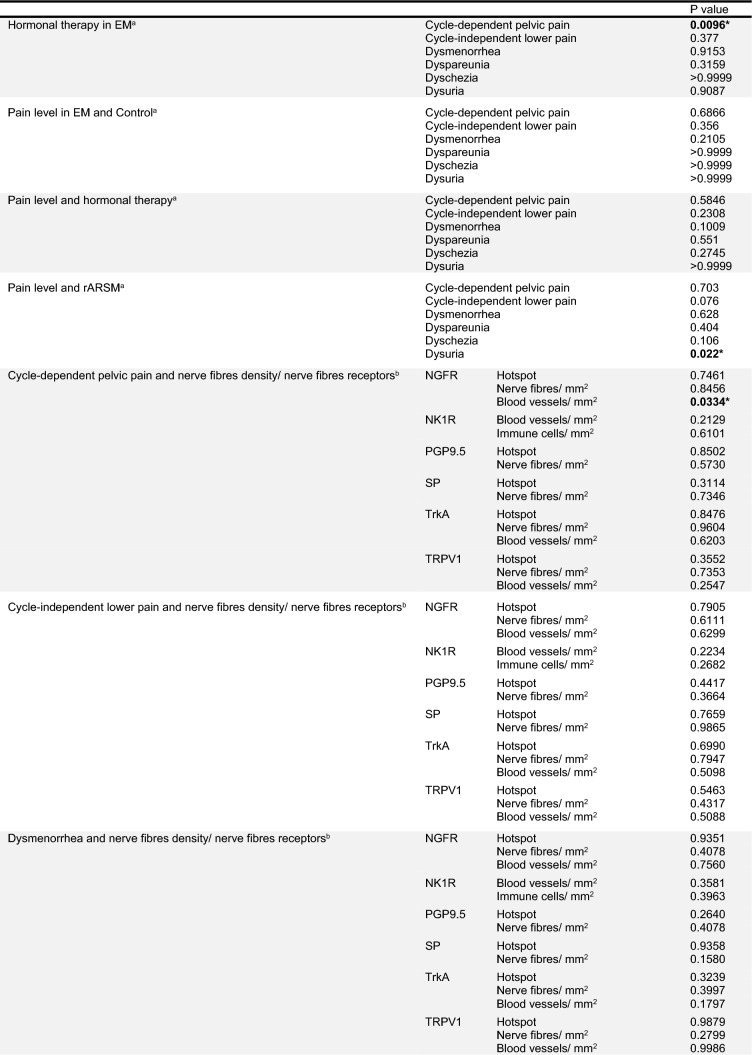

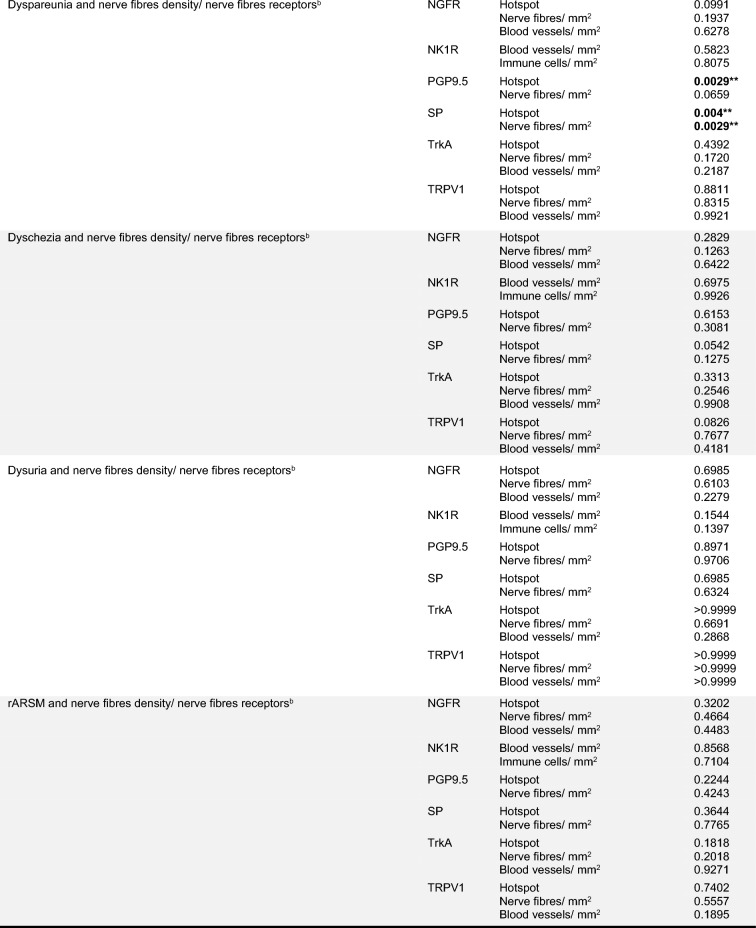
Correlation analysis

Bold values highlight the significant correlations

Analyses were made with ^a^*χ*^2^ or Fisher and ^b^Spearman correlation

*p < 0.05; **p < 0.005

#### Increased expression of NK1R in blood vessels and immune cells of EM patients

**Fig. 3:**
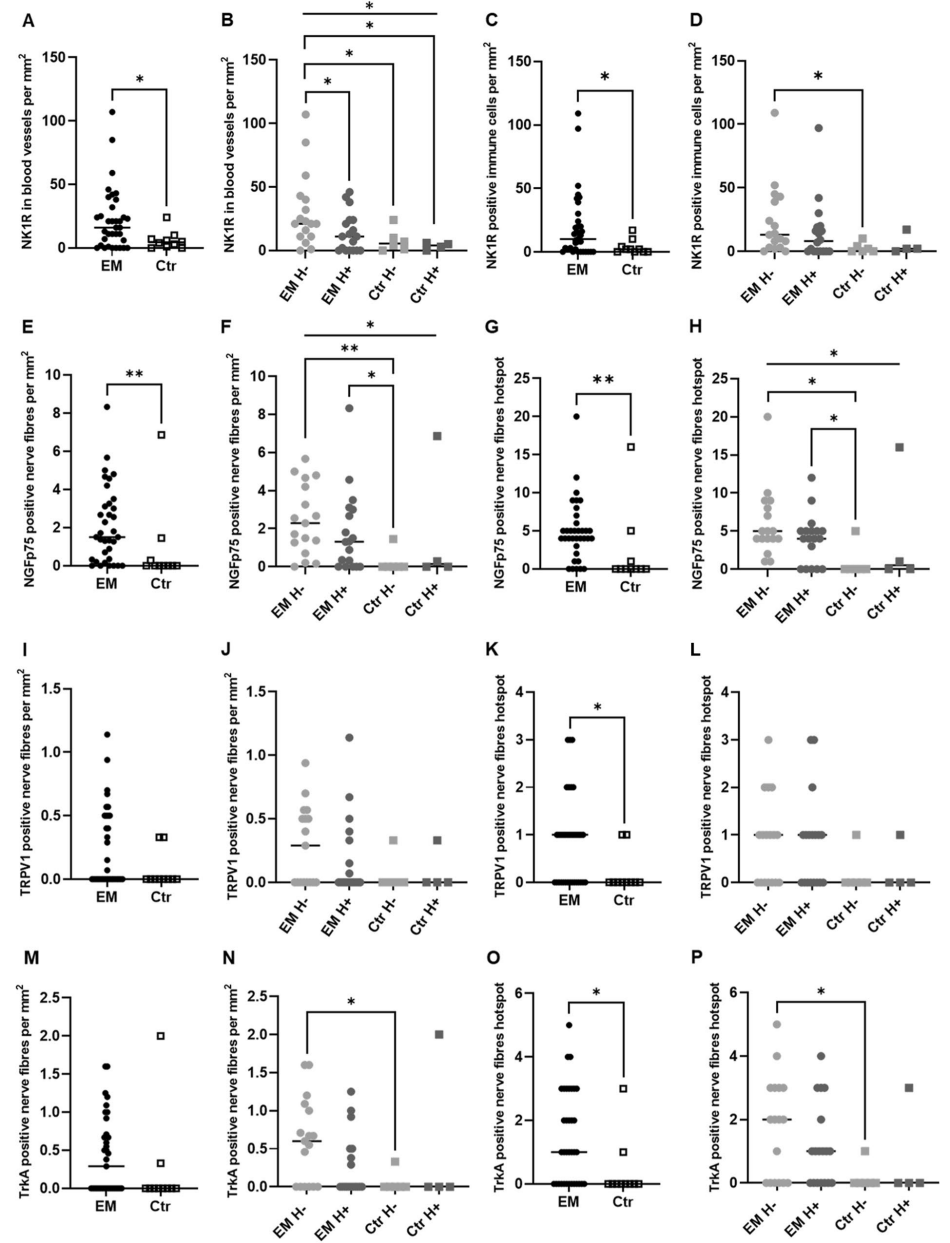
Endometriosis patients showed increased expression of nociceptive markers. NK1R-positive nerve fibers per mm^2^ (**A**–**B**) and hotspot (**C**–**D**); NGFp75-positive nerve fibers per mm^2^ (**E**–**F**) and hotspot (**G**–**H**); TRPV1-positive nerve fibers per mm^2^ (**I**–**J**) and hotspot (**K**–**L**); and TrkA-positive nerve fibers per mm^2^ (**M**–**N**) and hotspot (**O**–**P**). *EM* endometriosis patients, *Crt* control, *H +*  under hormonal treatment, *H*− without hormonal treatment; all the results are presented as median, 25–75% percentile. Mann–Whitney test and Kruskal–Wallis with Dunn’s multiple comparison tests. *p < 0.05; **p < 0.01; ***p < 0.001

EM patients presented more NK1R-positive stained vessels than the control group (EM: 16.0, 2.0–32.0; Ctr: 4.5, 0.75–7.75; *p* = 0.0302) (Fig. 3A). A statistical difference could also be found when the treatment was taken into account. EM patients under hormonal therapy showed fewer NK1R-positive stained vessels compared with EM patients without treatment (EM H + : 11.0, 0.0–24.0; EM H−: 21.0, 11.0–41.5; *p* = 0.0465) (Fig. 3B). Also, the EM group of patients without treatment differ from the control group (*p* = 0.0025), a difference that was not maintained when the EM patients with hormonal therapy were evaluated (*p* = 0.3312) (Fig. 3B). Regarding immune cells NK1R-positive, EM patients also have an increased amount of positive cells compared with the controls (EM: 10.0, 0.0–24.0; Ctr: 2.0, 0.0–5.5; *p* = 0.0415) (Fig. 3C). This statistical difference was maintained when EM without treatment was compared with controls also without hormonal therapy (EM H−: 13.0, 3.0–41.0; Ctr H−: 1.0, 0.0–5.5; *p* = 0.0184) (Fig. 3D).

No correlation could be seen between the NK1R expression and the rASRM stages. The NK1R-positive stained vessels and immune cells also did not correlate with the pain levels (Table 2).

#### EM patients showed increased expression of nociceptive markers

NGFRp75 staining showed a significant difference between EM patients and the control group when looking at the hotspot (EM: 4.0, 3.0–6.0; Ctr: 0.0, 0.0–5.0, *p* = 0.0064) and nerve fibers per mm^2^ (EM: 1.5, 0.33–3.26; Ctr: 0.0, 0.0–0.58; *p* = 0.0067) (Fig. 3E and G). EM patients under hormonal therapy and without also presented increased NGFRp75-positive nerve fibers in the hotspot (EM H + : 4.0, 0.0–5.0; EM H−: 5.0, 4.0–8.5; Ctr H−: 0.0, 0.0–1.25) and per mm^2^ view (EM H + : 1.31, 0.0–3.0; EM H−: 2.29, 0.98–4.43; Ctr H−: 0.0, 0.0–0.36) when compared with controls without treatment (hotspot *p* = 0.0429 and 0.0017; mm^2^ *p* = 0.0285 and 0.0011) (Fig. 3F and H). No difference was observed in the NGFRp75 stained vessels (data not shown).

When looking at TRPV1-positive colored nerves, EM patients showed more nerve fibers than the control group in the hotspot view (EM: 4.0, 3.0–6.0; Ctr: 0.0, 0.0–2.0; *p* = 0.039) (Fig. 3E), but not when looking at positively colored fibers per mm^2^ (EM: 1.5, 0.33–3.26; Ctr: 0.0, 0.0–0.58; *p* = 0.7520) (Fig. 3I and K) or taking into consideration the hormonal treatment (Fig. 3J and L). The analysis of TRPV1-stained vessels also showed no significant difference between these two groups (data not shown).

Women with EM showed an increased amount of TrkA colored nerve fibers compared to the control patients when looking at the hotspot view (EM: 1.0, 0.0–3.0; Ctr: 0.0, 0.0–0.25; *p* = 0.0242) (Fig. 3O), which was not the case when the nerve fibers per mm^2^ (*p* = 0.1079) (Fig. 3M) and the stained blood vessels (data not shown) were analyzed. In addition, a statistical difference was only obtained between EM and control patients without hormonal treatment for the hotspot (EM H−: 2.0, 0.0–3.0; Ctr H−: 0.0, 0.0–0.25; *p* = 0.0313) and nerve fibers per mm^2^ (EM H−: 0.6, 0.0–1.04; Ctr H−: 0.0, 0.0–0.08, *p* = 0.0234) (Fig. 3N and P).

No correlation could be seen between NGFRp75, TRPV1, and TrkA expression and the rASRM stages. However, a correlation between NGFRp75 stained blood vessels and the severity of the cycle-dependent pelvic pain was observed (*r* = − 0.5398; *p* = 0.0334) (Table 2).

## Discussion

The majority of research into the mechanisms underlying pain in EM has focused on the endometriotic lesions with the estrogen-dependent cyclical release of pain mediators as the primary source of EM-associated pain [10, 15]. This reflects the typical nociceptive pain, which disappears with the end of the menstrual bleeding and is the reason why hormonal treatment and nonsteroidal anti-inflammatory substances (NSAP) work, especially at the beginning of the disease. However, not seldom do patients experience a shift from cyclical to more acyclical pain or develop acyclical pain under hormonal treatment. This suggests the involvement of additional complex mechanisms.

Although lesion-specific pain is undoubtedly essential for the induction of EM-associated pain, lesion removal does not provide pain relief in all cases [7]. Furthermore, only a marginal association exists between lesion size or disease stage and the severity of pelvic symptoms [16]. A more recent understanding of the mechanisms underlying the development of a chronic pain state in EM implicates cyclical bleeding from lesions and subsequent inflammation at both lesion sites and in the peritoneal cavity. These proinflammatory responses then result in sensory nerve activation and altered activation of nociceptive pathways The complexity of peripheral and central sensitization makes research in this field very difficult.

In this study, we focused on peripheral sensitization of EM-associated nerve fibers: we investigated (i) the nerve fiber density of sensory nerve fibers in symptomatic EM patients, (ii) analyzed the expression of the SP and their receptor NK1R and (iii) the expression of nociceptive receptors and compared the findings between EM patients and controls and between patients using or not using hormonal treatment.

We demonstrated the presence of sensory nerve fibers in peritoneal endometriotic lesions in 45 women with confirmed symptomatic EM. The density of nerve fibers in peritoneal endometriotic lesions was much greater than in normal peritoneum in women with no EM, both in nerve fibers per mm^2^ and in hotspot image. EM patients also showed significantly more SP-positive nerve fibers in the hotspot view. Taken together, our data confirm the high innervation of the endometriotic lesion already seen by different groups [17–20]. These sensitive nerve fibers typically function as nociceptors, implicating them strongly in the generation of EM-associated pelvic pain [21, 22]. This supports the correlation between dyspareunia and the density of nerve fibers using anti-PGP9.5 and anti-SP seen in this study. The higher nerve fiber density goes in line with higher sensitivity in the case of mechanically stretching of the tissue during intercourse. There was no difference in the nerve fiber density between patients with and without hormonal treatment.

Neurogenic inflammation is caused by releasing the neurotransmitters from sensitive nerve endings, through interaction with immune cells [15] and might be the main source for a shift from cyclical to acyclical pain, or a reason for the development of acyclical pain under hormonal treatment. The expression of NK1R (receptor for SP) is reported to be upregulated by estrogen and TNF-α [19]. As local production of TNF-α and estrogen is increased in endometriotic lesions, NK1R expression would be and has been reported to be elevated [23]. In our study, NK1R could be detected in blood vessels but also immune cells at higher levels when compared with control patients. NK1R activation is involved in ERK1/2 protein (MAPK), p38 MAPK, NF-κB, PI3K, Akt, Src, EGFR and Rho/Rock signaling pathways in different cell types [24]. Importantly, all these proteins have been implicated in the development of EM [19]. To the best of our knowledge, this is the first study demonstrating this finding in peritoneal endometriotic lesions and gives the strong hint for evidence of neurogenic inflammation due to EM-associated nerve fibers.

Increased levels of neurotrophins such as NGF and their receptors NGFRp75 and TrkA are also seen in endometrial biopsies of women with EM [17, 25]. The greatly increased expression of NGFRp75 and TrkA by endometriotic lesions may also play a role in inducing the ingrowth of nerve fibers into endometriotic tissue and may play a primary role in setting up the mechanisms for the generation of pain [18, 26, 27]. These effects are exacerbated by increased levels of circulating estrogen in EM patients, as estrogen can enhance NGF activation of NGFRp75 and TrkA [28]. This is important as its downstream target is the well-known nociceptive cation channel TRPV1. The TRPV1 receptor is the most important activator of silent C-fibers. It was found to be upregulated in endometriomas and ectopic endometrial cells [29, 30], as well as in our EM samples suggesting the peripheral sensitization of the nerve fibers. This was, in all symptomatic patients, upregulated, independent from the use of hormonal treatment. An increase in the density of nerve endings throughout lesions and enhanced excitability of nerves provide the basis for increased nociception at lesion sites [10].

Pain severity was assessed to determine if there could exist a correlation between nerve fiber density, receptors expression and hormonal therapy. EM is associated with sexual pain, specifically, pain with deep penetration (dyspareunia). The etiology of dyspareunia in EM seems to be multifactorial [31], but a higher density of nerve fiber bundles around the endometriotic lesion, compared to patients without dyspareunia, was already confirmed [32]. Now, we show a correlation between this pain severity and the density of the nerve fibers. Along these lines, we indicate the correlation between NGFRp75 expression in blood vessels and the cycle-dependent pelvic pain severity. As commented above, this effect is aggravated by increased levels of estrogen in EM patients, as estrogen can enhance NGFRp75 activation [28].

**Fig. 4:**
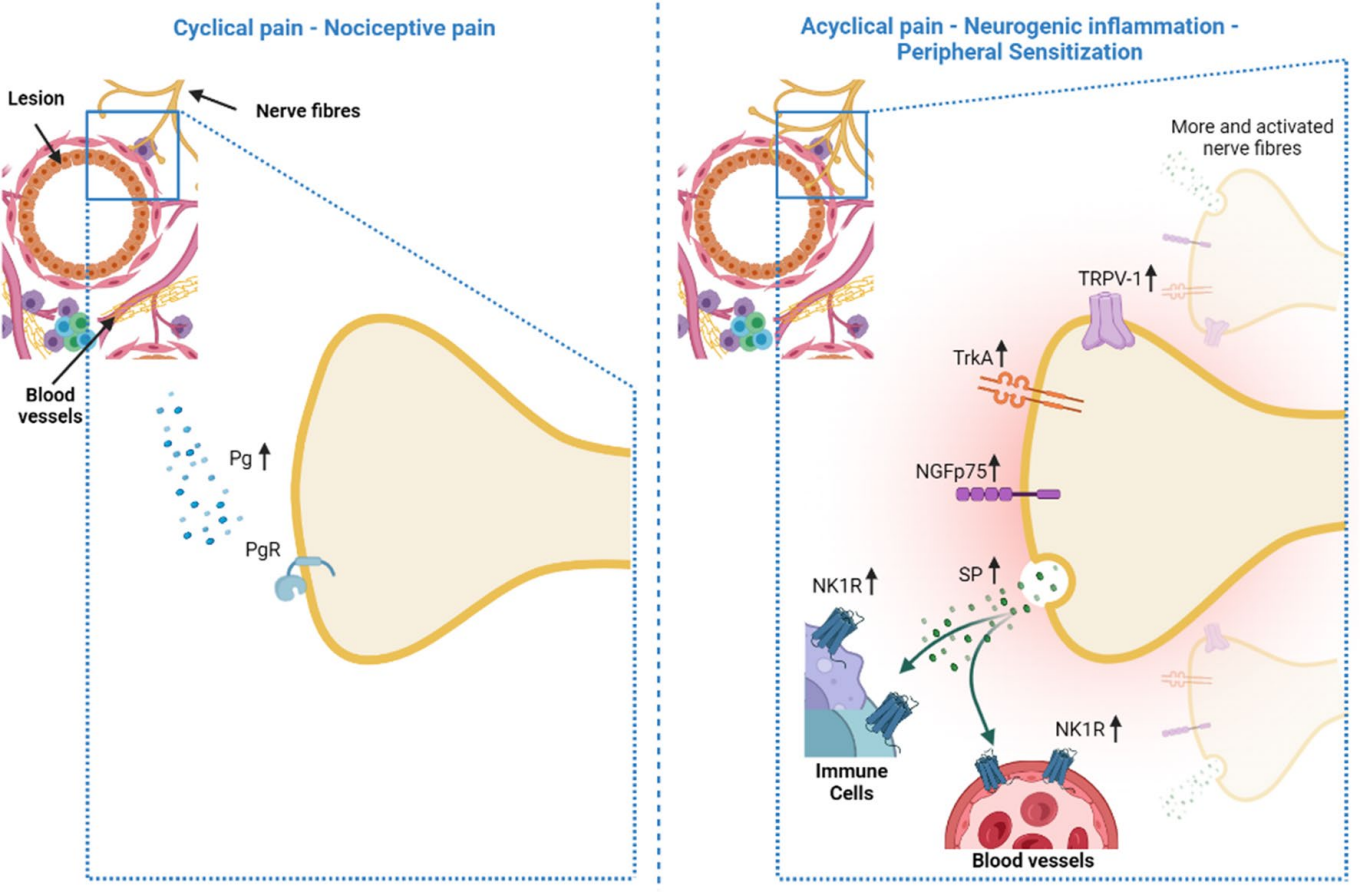
Summary of the cyclical and acyclical pain expression of nociceptive markers. In the cyclical pain (left), only the prostaglandin (Pg) and their receptor (PgR—prostaglandin receptor) are involved in the pain, in the nociceptive pain. In the acyclic pain (right), we have more and activated nerve fibers, increased expression of TRPV-1, TrKA, NGFp75 in the nerves, increased release of substance P (SP) and increased expression of NK1R in immune cells as well as in blood vessels

It has been shown that traditional hormone therapies that alleviate EM-associated pain, including progestogens and oral contraceptives, significantly reduced nerve fiber density in ectopic endometrium [33]. What we observed was a reduction of the cycle-dependent pelvic pain in EM patients to the treatment and the marginally reduced expression of NK1R in the blood vessels of these patients compared to those that did not receive the hormonal therapy. Since patients under hormonal therapy do not ovulate or bleed, makes sense that they also have no or less cyclic pain. As NK1R is related to inflammation (vasodilatation and interleukins release induction) [34], this makes us hypothesize that the hormonal intake is efficient only against the inflammation due to the EM but not to the pain itself as the other markers did not decrease with the treatment and the patients still suffer from acyclical pain (Fig. 4). This shows that neurogenic inflammation is present, and therefore causes the peripheral sensitization of the sensory nerve fibers.

Patients under hormonal therapy have no ovulation and no (menstrual) bleeding, which are typically associated with inflammation and cyclical pain. However, acyclical pain seems to be due to peripheral sensitization once it is present under the treatment. Neurotransmitters, like SP and their receptors, are involved in mechanisms of neurogenic inflammation, which are relevant for pain initiation in women affected by this chronic disease. Taking together, these findings seem to indicate that in both groups (EM with/without hormonal treatment), neurogenic inflammation is present and responsible for acyclical painful symptoms.

## Supplementary Information

Below is the link to the electronic supplementary material.

Supplementary file1 (DOCX 13 KB)

## Data Availability

Data available on reasonable request.
