# Supplementary material for: Mechanisms of peripheral sensitization in endometriosis patients with peritoneal lesions and acyclical pain
Source: Arch Gynecol Obstet. 2023 Jul 5;308(4):1327–40. doi: 10.1007/s00404-023-07110-9 (PMC10435658; doi:10.1007/s00404-023-07110-9)
Supplement: Supplementary file 1 — Supplementary file1 (DOCX 13 KB) [file 404_2023_7110_MOESM1_ESM.docx]

**Supplemental Table I. List of the antibodies and dilutions used in this study.**

| **Marker/ Receptor Antibody** | **Dilution** | **Company** |
| --- | --- | --- |
| Polyclonal rabbit anti-PGP9.5 (Protein gene product 9.5 ) | 1:1000 | Dako (Z5116) |
| Polyclonal rat anti-SP (Substance P) | 1:100 | Santa Cruz (sc-21715) |
| Polyclonal rabbit anti-NK1R (Neurokinin-1 Receptor) | 1:250 | Santa Cruz (sc-15323) |
| Monoclonal maus anti-NGFp75 (Nerve Growth Factor Receptor p75) | 1:1000 | Santa Cruz (sc-271708) |
| Polyclonal rabbit anti-TRPV1 (Transient Receptor Potential Vanilloid 1) | 1:200 | Alomone Labs (AC030) |
| Monoclonal rabbit anti-TrkA (Tropomyosin Receptor Kinase A) | 1:100 | Abcam (ab76291) |
